# Assessing the quality of anti-malarial drugs from Gabonese pharmacies using the MiniLab^®^: a field study

**DOI:** 10.1186/s12936-015-0795-z

**Published:** 2015-07-15

**Authors:** Benjamin J Visser, Janneke Meerveld-Gerrits, Daniëlle Kroon, Judith Mougoula, Rieke Vingerling, Emmanuel Bache, Jimmy Boersma, Michèle van Vugt, Selidji T Agnandji, Harparkash Kaur, Martin P Grobusch

**Affiliations:** Division of Internal Medicine, Department of Infectious Diseases, Center of Tropical Medicine and Travel Medicine, Academic Medical Center, University of Amsterdam, Meibergdreef 9, PO Box 22700, 1100 DE Amsterdam, The Netherlands; Centre de Recherches de Médicales de Lambaréné (CERMEL), Albert Schweitzer Hospital, Lambaréné, Gabon; Institute of Tropical Medicine, University of Tübingen, Tübingen, Germany; Department of Pharmaceutical Technology and Biopharmacy, Utrecht University, Utrecht, The Netherlands; Department of Clinical Research, London School of Hygiene and Tropical Medicine, London, UK

**Keywords:** Artemisinin combination therapy (ACT), Central Africa, Counterfeit, Falsified, Field survey, Gabon, Malaria, Medicine quality, MEDQUARG, Sub-standard

## Abstract

**Background:**

Recent studies alluded to the alarming scale of poor anti-malarial drug quality in malaria-endemic countries, but also illustrated the major geographical gaps in data on anti-malarial drug quality from endemic countries. Data are particularly scarce from Central Africa, although it carries the highest burden of malaria. The aim of this medicine quality field survey was to determine the prevalence of poor-quality anti-malarial drugs in Gabon.

**Methods:**

A field survey of the quality of anti-malarial drugs in Gabonese pharmacies was conducted using the Global Pharma Health Fund Minilab^®^ tests, following the Medicine Quality Assessment Reporting Guidelines. Anti-malarial drugs were purchased randomly from selected pharmacies in Gabon. Semi-quantitative thin-layer chromatography (TLC) and disintegration testing were carried out to measure the concentration of active pharmaceutical ingredients (APIs). The samples failing the TLC test were analysed by high-performance liquid chromatography. Following the collection of anti-malarial drugs, a street survey was conducted to understand where people purchase their anti-malarial drugs.

**Results:**

A total of 432 samples were purchased from 41 pharmacies in 11 cities/towns in Gabon. The prevalence of poor-quality anti-malarial drugs was 0.5% (95% CI 0.08–1.84%). Two out of 432 samples failed the MiniLab^®^ semi-quantitative TLC test, of which a suspected artemether-lumefantrine (AL) sample was classified as falsified and one sulfadoxine-pyrimethamine (SP) sample as substandard. High performance liquid chromatography with ultraviolet photo diode array detection analysis confirmed the absence of APIs in the AL sample, and showed that the SP sample did contain the stated APIs but the amount was half the stated dose. Of the people interviewed, 92% (187/203) purchased their anti-malarial drugs at a pharmacy.

**Conclusion:**

Using the GPHF Minilab^®^, the prevalence of poor-quality anti-malarial drugs is far lower than anticipated. The findings emphasize the need for randomized and robust sampling methods in order to collect representative data on anti-malarial drug quality.

Trial registration: NTR4341 (Dutch Trial Registry)

**Electronic supplementary material:**

The online version of this article (doi:10.1186/s12936-015-0795-z) contains supplementary material, which is available to authorized users.

## Resume

**Contexte:**

Des études récentes ont fait allusion à l'ampleur alarmante sur la qualité des médicaments antipaludiques dans les pays d'endémie. Elles ont aussi illustré les principales lacunes des données géographiques concernant les médicaments antipaludiques dans les pays endémiques. Les données sont particulièrement rares en Afrique centrale, bien que cette région porte le plus lourd fardeau du paludisme. Le but de cette enquête sur le terrain était de déterminer la prévalence des médicaments antipaludiques de mauvaise qualité au Gabon.

**Méthodes:**

Une enquête sur le terrain a été réalisée sur la qualité des médicaments antipaludiques dans les pharmacies gabonaises en utilisant les tests ‘Global Pharma Health Fund Minilab®’, suivant la ‘Medicine Quality Assessment Reporting Guidelines (MEDQUARG)’. Les médicaments antipaludiques ont été achetés dans des pharmacies choisies au hasard au Gabon. La chromatographie semi-quantitative en couche mince (CCM) et le test de désintégration ont été effectués pour mesurer la concentration d'ingrédients pharmaceutiques actifs (API). Les échantillons qui n’ont pas réussi le test CCM ont été analysés par chromatographie en phase liquide à haute performance. Après la collecte des médicaments antipaludiques, une enquête communitaire a été menée pour comprendre où les gens achètent leurs médicaments.

**Résultats:**

Un total de 432 échantillons ont été achetés dans 41 pharmacies de 11 villes au Gabon. La prévalence de médicaments antipaludiques de mauvaise qualité était de 0,5% (IC 95% de 0,08 à 1,84%). Sur les 432 échantillons, le test semi-quantitatif de chromatographie sur couche mince Minilab® n’a permis de détecter aucune substance active sur deux échantillons, dont un échantillon d’artéméther-luméfantrine (AL) suspecté contrefait et un échantillon de sulfadoxine-pyriméthamine (SP) ont été classées échantillon de qualité inférieure. L’analyse pour la détection d’APIs dans l’échantillon AL par chromatographie liquide à haute performance avec photo ultraviolet de réseau de diodes a confirmé l'absence d'API et a montré que l'échantillon de SP contient les énoncés API mais la quantité était la moitié de la dose indiquée. Parmi les personnes interrogées, 92% (187/203) ont acheté leurs médicaments dans une pharmacie.

**Conclusion:**

L'utilisation du GPHF Minilab®, la prévalence des médicaments antipaludiques de mauvaise qualité est grandement inférieur à celle prévue. Les résultats soulignent la nécessité pour les méthodes d'échantillonnage aléatoire et robustes afin de recueillir des données représentatives sur la qualité des médicaments antipaludiques.

## Background

*Plasmodium falciparum* malaria is estimated to cause 528,000 deaths and 163 million clinical episodes in Africa [1]. Early diagnosis and treatment with appropriate anti-malarial drugs can prevent severe illness and lethal outcome [2–4]. Therefore, it is crucial that the administered anti-malarial drugs are of acceptable quality [5]. In Gabon, the majority of anti-malarial drugs are purchased directly by the patient or caretaker from the pharmacy (licensed and unlicensed) for self- or home treatment. There is no anti-fake medicine programme, nor an effective drug regulatory system in Gabon (Additional file 1). Gabon does not receive international donor support for anti-malarial medicines. The national malaria control programme of Gabon does not provide anti-malarials for free. Whether quality assured or falsified, anti-malarial drugs have not been reported from the Gabonese markets as from the neighbouring countries. The spread of poor-quality [6, 7] (e.g., counterfeit or falsified) anti-malarial drugs may pose an obstacle to effective malaria control. Poor-quality anti-malarial drugs have serious consequences for public health [5]. Drugs with too little, or devoid of active pharmaceutical ingredients (APIs) may cause increased morbidity and mortality [8]. Also, low concentrations of APIs in poor-quality drugs will result in sub-therapeutic concentrations of the drug in vivo, which may contribute to the selection of resistant parasites [9]. Furthermore, the use of poor-quality anti-malarial drugs leads to financial loss for patients and their families, healthcare systems and pharmaceutical companies producing the genuine product [10]. The general public can lose confidence in a pharmaceutical brand, drugs, pharmacies, and healthcare providers [11].

A systematic review in 2014 illustrated the alarming scale of poor anti-malarial drug quality in malaria-endemic countries, but also showed major geographical gaps, with no published information on the quality of anti-malarial drugs from 60.6% (63/104) of the malaria-endemic countries) [12]. Using the Worldwide Antimalarial Resistance Network (WWARN) [13] database, it was demonstrated that out of 9,348 anti-malarial drugs collected (compiled from 130 publications in total), 30.1% (2,813) failed chemical/packaging quality tests with 39.3% classified as falsified; 2.3% as sub-standard and 58.3% as poor-quality, without evidence available to categorize them as either sub-standard or falsified [12]. There are few reports originating from Central Africa. Also for Gabon, systematic data on the geography and epidemiology of poor-quality anti-malarial drugs is scarce. Gabon is a high-endemicity country for malaria [14–16]. A study in 2011, assessing the quality of chloroquine tablets in 12 African countries collected two chloroquine samples from the capital of Gabon (Libreville), which were both of good quality [17]. The World Health Organization (WHO) investigation in 2003 collected 25 chloroquine samples (29% poor-quality) and ten sulfadoxine-pyrimethamine samples (100% good quality) from pharmacies in Libreville. A limited number of reports are available from neighbouring countries Cameroon [5, 17–22], Equatorial Guinea [23] and the Republic of Congo [5].

The aim of this study was to determine the prevalence of poor-quality anti-malarial drugs in Gabon, which lacks an effective national product quality monitoring programme (see Additional file 1). Information about the quality of anti-malarial drugs is important for improving malaria treatment and to successfully run malaria control programmes [24, 25].

## Methods

### Registration and reporting

This medicine quality field survey was registered in advance (30 Dec 2013) in The Netherlands Trial Registry (NTR): NTR4341 [26]. This report follows, where appropriate, the Medicine Quality Assessment Reporting Guidelines (MEDQUARG) [27, 28]. Also, the costs of this study are reported [29] (Additional file 2).

### Scientific research and ethical committee statement

Scientific clearance was obtained from the Scientific Review Committee (SRC) of the Centre de Recherches de Médicales de Lambaréné (CERMEL), Albert Schweitzer Hospital (SRC number: 2013.11; Additional file 3). The Ethical Committee of CERMEL decided that ethical approval of this study was not required as this study is a quality assurance in healthcare study, no humans having been subjected to it [30].

### Study area

Gabon (an upper-middle income country, GDP $19.34 billion, 2013) straddles the Equator. About 80% of its 267,667 km^2^ area is covered by dense tropical rainforest. The population of Gabon is estimated to be around 1.6 million inhabitants (6.3 inhabitants/km^2^), 86.2% of whom live in urban areas. CERMEL is based in Lambaréné, the capital of the Moyen-Ogooué Province, a semi-urban town of about 30,000 inhabitants surrounded by villages. Gabon is administratively divided into nine provinces, with villages mainly located along roads and rivers. Gabon is a highly malaria-endemic country. The official first-line treatment for uncomplicated falciparum malaria is artesunate + amodiaquine (AS + AQ) [31] or artemether-lumefantrine (AL) and severe falciparum malaria is treated with intravenous quinine. Intramuscular use of artemether or intravascular artesunate is not common in Gabon.

### Primary and secondary outcomes

The primary outcome was the proportion (percentage) of poor-quality anti-malarial drugs in pharmacies in Gabon. Secondary outcomes were the proportion of outlets selling poor-quality anti-malarial drugs and availability of anti-malarial drugs that are no longer recommended as first- or second-line treatment in Gabon or by WHO. The following secondary outcome was added during the study to assess the external validity of the field survey: to determine where people purchased their anti-malarial drugs.

### Definitions

The overarching term ‘poor-quality drugs’ is used to describe the different categories: falsified medicines are fake medicines that are designed to mimic real medicines; counterfeit medicines are medicines that do not comply with intellectual-property rights or that infringe trademark law.

### Timing and location of the survey

**Figure 1:**
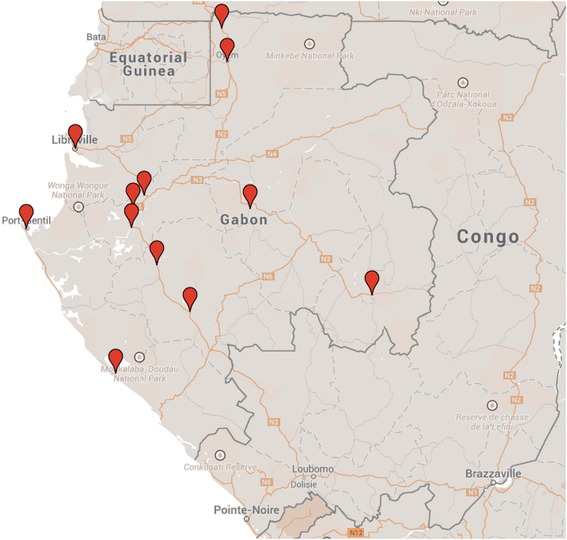
Map of sampling sites in Gabon (Google Maps).

The field survey was conducted in January 2014 in Gabon. The six (out of nine) most populated provinces (ISO 3166-2:GA) were selected: Estuaire; Haut-Ogooué, Moyen-Ogooué, Ngounié, Ogooué-Maritime, and Woleu-Ntem. Selected locations were: Libreville (capital), Franceville, Lambaréné, Mouila, Port-Gentil, Oyem, Bitam, Owendo, Fougamou, Makouke, Bifoun, Gamba, and Lopé (Figure 1).

### Sampling design and sample size

There are approximately 75 open and fully functioning pharmacies in Gabon (2013) [32]. Pharmacies were randomly selected. The randomization procedure was performed by BJV using statistical software (nQuery Advisor^®^ Version 7.0. Statistical Solutions, Cork, Ireland) on the day before the actual sampling. A full list provided by the Health Authorities of Gabon and a list from the National Health Assurance Company (La Caisse Nationale d’Assurance Maladie et de Garantie Sociale du Gabon [33], CNAGMS) with registered/licensed pharmacies and dispensaries was prepared (before sampling) to allow for proper randomization procedures. This list was accomplished with (unlisted) pharmacies by local nurses and fieldworkers. In total, six pharmacies were found which were missing on the CNAGMS list of pharmacies in Gabon. In Lambaréné (Moyen-Ogooué Province), where the CERMEL is based, all known pharmacies (n = 7) were sampled during the first week of the sampling period and thus not randomly selected. In all other areas, approximately 50% of the pharmacies were randomly selected in all neighbourhoods. Hospitals (except for the Albert Schweitzer Hospital), markets, grocery shops, and street peddlers were not visited because regulations for drug selling are in place; previsits by local nurses yielded little evidence (if any) of anti-malarial drugs sold there. The appropriate sample size and strategy is challenging, since data of the prevalence of poor-quality anti-malarial drugs are very scarce for Gabon. Thus, the most conservative sample size is given by using an unknown prevalence [hypothesized 50% frequency of outcome factor in the population (p)]. To determine the actual prevalence of poor-quality drugs available in Gabon with a precision of 5% with 95% confidence intervals (z = 1.96), a random sample of 384 was needed. The following equation was used: sample size $n = \, \left[ {{\text{DEFF}}*{\text{Np}}\left( {1 - {\text{p}}} \right)} \right]/ \, [({\text{d}}^{2} /{\text{Z}}^{2}_{1 - \alpha /2} )*\left( {{\text{N}} - 1} \right) + {\text{p}}*\left( {1 - {\text{p}}} \right)].$ The sample size was calculated with OpenEpi (Open Source Epidemiologic Statistics for Public Health) version 3.03 [34].

### Sampling procedure

A Gabonese ‘mystery shopper’ (JM, nurse, of Gabonese nationality) conducted the actual sampling process in Moyen-Ogooué (177/432 samples) and was trained utilizing standard sampling guidelines. She dressed according to regular Gabonese standards and gave no indication that she was not a regular shopper. A standard scenario was used: she asked which anti-malarial drugs were for sale. Subsequently, she purchased one full child/adult treatment in their original packaging of each of the available anti-malarials and of each available brand, but not of each available batch. Samples included drugs sold in the manufacturer’s original packaging as well as those distributed loose, often in plastic bags. Surprisingly, sellers never asked questions. Although the US Pharmacopeia (USP) recommends 30 dosage units [35] for a single tablet of the same lot number from each location, this was not deemed practically feasible in the Gabonese setting, and also too expensive. For logistical reasons the other 255/432 (60%) samples were collected by the investigators. Anti-malarial drugs purchased included: AL, AS + AQ, AS + sulfadoxine, AS-mefloquine, dihydroartemisinin-piperaquine, dihydroartemisinin-piperaquine-trimethoprim, artemisinin-piperaquine, artemisinin-naphtoquine, quinine, sulfadoxine-pyrimethamine (SP), mefloquine, proguanil, atovaquone-proguanil, proguanil-chloroquine, pyrimethamine and chloroquine. Other anti-malarial drugs were not purchased. Only solid dosage forms were collected (no liquid formulations). To avoid potential bias in subsequent sampling rounds, the exact reason for sampling medicines was not shared with the seller. Results were not reported back to the seller. For every sample collected, the collector completed and signed the sample collection form (including GPS locations) (Additional file 4) as soon as possible after leaving the point of sale and before performing the next purchase. Once purchased, all drugs were stored until testing at room temperature (in an air-conditioned room) with no sunlight. Humidity could not be controlled. Tests were completed at the laboratory of the Academic Medical Centre (AMC, The Netherlands) within 3 months of sample collection.

### Questionnaire

To determine where people purchased their anti-malarial drugs, a questionnaire was presented to food market dwellers in Lambaréné as well as at PK (‘point kilomètre’) 8 *Le Marché Bananes* (a transportation hub) and *Marché du Mont*-*Bouët* in Libreville. The survey was conducted after the purchase of the anti-malarial drugs. The most important question in this survey was: “where do you buy anti-malarial drugs?”.

### Storage and shipment of samples

Before shipment by air, samples were stored at CERMEL under appropriate storage conditions. The samples arrived within 36 h at AMC in Amsterdam, The Netherlands. Samples were protected by appropriate packaging (primary container and additional packaging) during shipment by air.

### Chemical and packaging analysis

Samples were analysed at the Internal Medicine Research Laboratory of the AMC between February 2014 and April 2014 by BJV and JMG. The chemical analysis was performed unblinded to packaging. The Global Pharma Health Fund (GPHF) Minilab^®^ (Merck Darmstadt, Germany) was used to run semi-quantitative thin-layer chromatography (TLC) and disintegration tests on each sample to determine the presence and relative concentration of APIs [36]. Expired drugs were also tested. The MiniLab^®^ protocols award products a ‘pass’ for TLC if 80% or more of the labelled active ingredient(s) is present. For fixed-dose combinations (e.g., AL) and SP, ‘pass’ was awarded only if both active ingredients met this standard. TLC is an accepted method to assess the quality of drugs [37, 38]. The MiniLab protocols have been reviewed by the Promoting the Quality of Medicines (PQM) programme operated by the USP Convention. Each sample/test was run twice on separate days (once by BJV, once by JMG), with the assumption that the result most consistent with the reference was recorded. Thus, every API in every sample was tested twice. Standard operating procedures (SOP) provided with the Minilab were used [36]. Quality control of the GPHF MiniLab^®^ was performed daily before the drug testing and consisted of performing TLC on Minilab-reference samples for the anti-malarial drug analysed. In addition, Minilab reagents were quality control tested, using reference samples when a new lot was introduced. Samples were also tested to see if they disintegrated in purified water, following the guidelines of the European Pharmacopeia (EP) [39]. For this, apparatus A as described in the EP 2.9.1. was used at the laboratory of the Pharmaceutical Technology and Biopharmacy Department of Utrecht University (Utrecht, The Netherlands). Since the disintegration test requires six tablets per test, not all samples could be analysed. To include as many samples as possible, it was decided to test the samples per batch number instead of per sample. All samples with the same batch (or LOT) number, were expected to be homogeneous [40]. Samples failing TLC were analysed using the high-performance liquid chromatography with ultraviolet photo-diode array detection (HPLC–UV-PDA [41] February 2015 in a reference laboratory at the London School of Hygiene and Tropical Medicine) to quantify the amount of APIs present in each sample. This was compared with the stated dose on the packaging and the spectra achieved using a quality assured sample. For artemisinin derivatives, the artemisinin derivative screening test (ADST) was conducted according to an earlier published method [38]. Not all samples were analysed by HPLC–UV-PDA (the gold standard) due to lack of funding.

The packaging analysis was developed in line with previously published research [5, 27]. For the packaging analysis, genuine samples were requested by email from the manufacturers using a standard letter; with two reminders sent 2 and 4 weeks after the first email. Unfortunately no genuine anti-malarial samples were received.

### Statistical analysis

Descriptive statistics were performed using SPSS 20.0 statistical package (SPSS Inc., Chicago, MI, USA). The confidence interval of the prevalence estimate was calculated using the Wilson procedure with a correction for continuity. Fisher’s exact test was used to calculate the difference between the number of poor-quality drugs of the Gabonese mystery shopper *versus* two European researchers. Inter-observer reliability (for chemical analysis) was calculated using the Kappa (κ) statistic [42].

### Sharing data with Medicine Regulatory Agency

The results of this field survey were shared with the Medicine Regulatory Agency (MRA) in Gabon, the Director of Health of the Province Moyen-Ogooué and Rapid Alert of the WHO.

## Results

The samples purchased in this field survey were readily available over the counter without prescription in all pharmacies. In total, 432 full anti-malarial treatments were collected from 41 pharmacies and one hospital (Albert Schweitzer Hospital, Lambaréné) in 11 cities/towns in Gabon (Table 1; Figure 1). From the collected data, 55% (41/75) of pharmacies in Gabon were surveyed. The ‘class’ of pharmacy and licensing status (e.g., public, private for profit, private not for profit, informal) was not determined and the drug sellers were not interviewed.

**Table 1:** Anti-malarial drugs collected

	Collected and analyzed with TLC (%)	Samples included in disintegration test (% of TLC tested)	Expired before analysis	TLC failures	Disinteg-ration test failures	Average costs (USD)
ACT						
Artemether-lumefantrine	177 (41%)	102 (58%)	3	1	0	7.6
Artesunate-amodiaquine	42 (10%)	22 (52%)	6	0	0	7.2
Artesunate-SP	36 (8%)	26 (72%)	1	0	0	8.5
Dihydroartemisinin-piperaquine	38 (9%)	33 (86%)	1	0	0	9.9
Artesunate-mefloquine	22 (5%)	5 (23%)	1	0	0	9.7
Dihydroartemisinin-piperaquine- trimethoprim	13 (3%)	12 (92%)	0	0	0	9.3
Dihydroartemisinin-SP	7 (2%)	0 (0%)	0	0	0	4.5
Artemisinin-piperaquine	2 (<1%)	0 (0%)	0	0	0	10.1
Artemisinin-naphtoquine	1 (<1%)	0 (0%)	0	0	0	NR
Other						
Quinine	43 (10%)	34 (79%)	0	1	0	9.6
Sulphadoxine-pyrimethamine	40 (9%)	26 (65%)	0	0	1	2.2
Mefloquine	4 (<1%)	2 (50%)	0	0	0	33.3
Atovaquone-proguanil	2 (<1%)	0 (0%)	0	0	0	24.2
Proguanil	2 (<1%)	0 (0%)	0	0	0	16.5
Proguanil-chloroquine	2 (<1%)	0 (0%)	1	0	0	25.2
Pyrimethamine	1 (<1%)	0 (0%)	0	0	0	NR
Total	432 (100%)	266 (62%)	13 (3%)	2	1	8.1

*TLC* semi-quantitative thin-layer chromatography.

Of the 432 collected samples, 338 (78%) were artemisinin-based combination therapy (ACT). AS + AQ, the national recommended first-line treatment for falciparum malaria, comprised 10% of the total samples (n = 42). The second-line anti-malarial drug combination is AL and the third-line drug combination is dihydroartemisinin-piperaquine. An ACT was available in every surveyed pharmacy, but AS-AQ was only available in 27 pharmacies (65%). On average, ten full anti-malarial treatments were collected per pharmacy (min–max: 3–20). Two samples were expired at the day of purchase, both collected in a pharmacy in Lambaréné. They were not classified as sub-standard. No chloroquine or artesunate monotherapy was for sale in all the surveyed pharmacies.

### Chemical analysis: semi-quantitative thin-layer chromatography (TLC)

**Figure 2:**
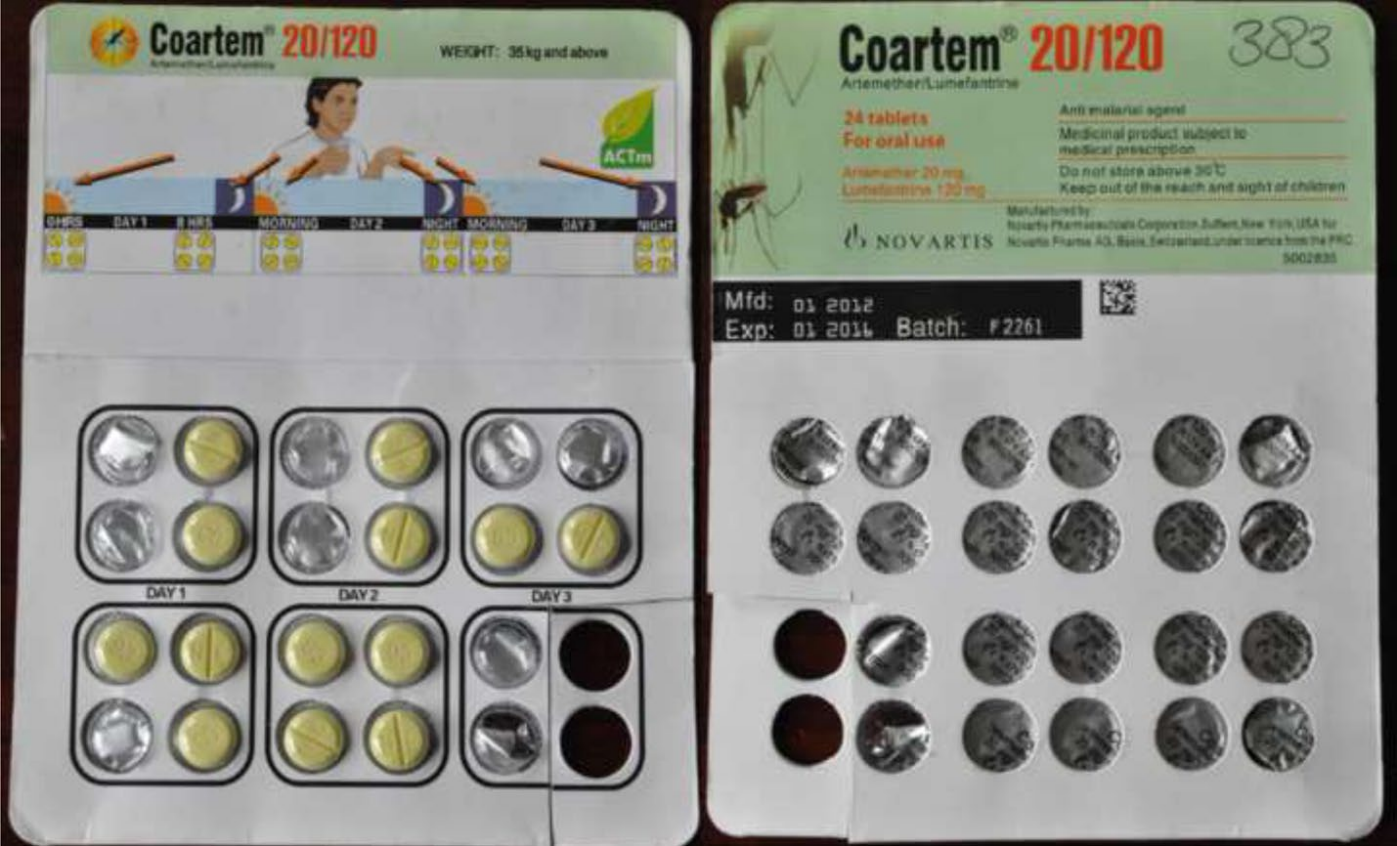
The suspect Coartem^®^ sample, having failed semi-quantitative thin-layer chromatography.

All samples were analysed in duplicate on separate days (Table 2). Inter-observer agreement was good for TLC testing (κ = 1, 100% agreement). Thirteen samples were expired before analysis, but analysed anyway since they were sold. All samples met the requirements for uniformity of UV spots (254 nm light), which meant that all samples were analysed correctly. Only two of the 432 (0.5%) samples analysed failed the TLC 0.5% (95% CI 0.08–1.84%). The first failed sample was AL 20/120 mg (Coartem^®^) (Figure 2), with batch number F2261, manufacture date 01.2012 and expiry date 01.2016. This sample was collected in a pharmacy in Bitam, a town 30 km south of the border with Cameroon. The genuine product is manufactured by Novartis and is a WHO pre-qualified medicine (Table 2). This sample did not contain any APIs. Storage conditions in Gabon or during transport are not expected to have been of any influence, given AL’s excellent stability profile in humid and hot conditions [43]. This batch number is also known to be falsified: in November 2013, a drug alert of the WHO was issued describing falsified batches of Coartem^®^ circulating in Cameroon [44]. The sample bears the falsified green leaf logo of the Affordable Medicines Facility—Malaria (AMFm) programme. There were actually made of calcium phosphates, fatty acids and yellow pigment, according to a copy of a Novartis analysis of the tablets reviewed by *The Wall Street Journal* [45, 46]. Another important clue for falsification is the interval between manufacturing and expiry date, which was 4 years in this sample, but should be 23 months.

**Table 2:** Summary of formulations tested with the brand name, manufacturer, dose, dosage form, ACT Watch Antimalarial database status and WHO prequalification list

Brand name/manufacturer	Generic name and dose	ACT Watch Antimalarial Database Antimalarial category	Regulatory status in Gabon
Alaxin-SP^®^, Bliss GVS Pharma LTD, India	Dihydroartemisinin + SP tablet 60/500/25 mg	Non-quality assured ACT	Unknown
Arco^®^, Kunming Pharmaceutical Corp, China	Artemisinin-naphthoquine tablet 125/50 mg	Non-quality assured ACT	Unknown
Arsiquinoforme^®^, Sanofi Aventis, Cote d’Ivoire	Quinine tablet 250 mg	Non-artemisinin therapy	Unknown
Artecom^®^, Tonghe Pharmaceutical Co. Ltd, China	Dihydroartemisinin-piperaquine-trimethoprim tablet 32/320/90 mg and 16/160/45 mg	Non-quality assured ACT	Unknown
Artedar^®^, Plethico Pharma Ltd, India	Artesunate-SP, tablet 100/500/25 mg	Non-quality assured ACT	Unknown
Artediam^®^, Adams Pharmaceutical (Anhui) Co., Ltd.	Artesunate-amodiaquine tablet 100/300 mg	Not listed, category unknown.	Approval at national level
Artefan^®^, Ajanta Pharma Ltd, India	Artemether-lumefantrine tablet 20/120 mg Artemether-lumefantrine tablet 80/480 mg	QAACT (Quality-assured ACT) Non-quality assured ACT	A (MA092, 2012-Dec-19)—WHO prequalification Approval at national level
Artequick^®^, Artepharm Co.Ltd, China	Artemisinin-piperaquine tablet 62.5/375 mg	Non-quality assured ACT	Unknown
Artequin^®^, Mepha Ltd, Switzerland	Artesunate-mefloquine tablet 200/250 mg Artesunate-mefloquine tablet 100/125 mg	QAACT (Quality-assured ACT) Non-quality assured ACT	B
Artim^®^, Twight Litaka Pharma Limited Ltd, India	Artemether-lumefantrine tablet 40/240 mg	Not listed, category unknown	Unknown
Artiz (Forte)^®^, Alice Pharma Pvt Ltd, India	Artemether-lumefantrine tablet 40/240 mg Artemether-lumefantrine tablet 20/200 mg	Dosage not listed, category unknown Dosage not listed, category unknown	Unknown
ASAQ Denk^®^, Denk Pharma, Germany	Artesunate-amodiaquine tablet 100/270 mg	Non-quality assured ACT	Unknown
Asunate Denk^®^, Denk Pharma, Germany	Artesunate-SP tablet 200/500/25 mg	Non-quality assured ACT	Unknown
Bimalaril^®^, Bengba Pharmaceutical factory, China	Artemether-lumefantrine tablet 80/480 mg	Not listed, manufacturer unknown	Approval at national level
Chinther^®^, Alkema Laboratories LTD, India	Artemether-lumefantrine tablet 40/240 mg	Not listed, brand and manufacturer unknown	Unknown
Co-Arinate^®^, Famar Italia Spa, Italy	Artesunate-SP tablet 200/500/25 mg Artesunate-SP tablet 100/250/12,5 mg	Not listed, manufacturer unknown Not listed, manufacturer unknown	Unknown
Coarsucam^®^, Sanofi Aventis, Morocco	Artesunate-amodiaquine tablet 50/135 mg Artesunate-amodiaquine tablet 100/270 mg Artesunate-amodiaquine tablet 25/67.5 mg	QAACT (Quality-assured ACT) QAACT (Quality-assured ACT) QAACT (Quality-assured ACT)	MA057 (2008-Oct-14)/MA058 2008-Oct-14/MA056 (2008-Oct-14) WHO prequalification
Coartem^®^, Novartis Pharma Ag, China, Switzerland, USA	Artemether-lumefantrine tablet 20/120 mg	QAACT (Quality-assured ACT)	A-B (MA026 (a), 2004-Apr-26) WHO prequalification/FDA approval
Co-artemax^®^, GA Pharma, Greece	Dihydroartemisinin-piperaquine tablet 40/320 mg	Not listed, brand & manufacturer unknown	Unknown
Cofantrine^®^, Bliss Gvs Pharma Ltd, India Cofantrine^®^, EGR Pharma, Luxembourg	Artemether-lumefantrine tablet 20/120 mg and 80/470 mg Artemether-lumefantrine tablet 20/120 mg	Non-quality assured ACT Not listed, unknown manufacturer	Approval at national level
Colart^®^, Glaxosmithkline Group Of Companies, India	Artemether-lumefantrine tablet 20/120 mg	Non-quality assured ACT	Unknown
Combimal^®^, Ajanta Pharma Ltd, India/Mauritius	SP tablet 500/25 mg	Non-artemisinin therapy	Unknown
Darte-Q^®^, Gosun Pharma Corp, China	Dihydroartemisinin-piperaquine tablet 40/320 mg	Non-quality assured ACT	Unknown
Duo-Cotecxin^®^, Beijing Holley-Cotec Pharmaceutical Ltd, China	Dihydroartemisinin-piperaquine tablet 40/320 mg	Non-quality assured ACT	Unknown
Falquin^®^, Plethico pharmaceutical Ltd, India	Quinine tablet 300 mg.	Not listed, manufacturer unknown	Unknown
Fansidar^®^, F.Hoffmann La Roche Ltd, Switzerland	SP tablet 500/25 mg	Non-artemisinin therapy	Unknown
Lariam^®^, F.Hoffmann La Roche Ltd, Switzerland	Mefloquine tablet 250 mg	Non-artemisinin therapy, country of manufacture not listed	Unknown
Laritem^®^, IPCA Laboratories Ltd, India	Artemether-lumefantrine 20/120 mg Artemether-lumefantrine 80/480 mg	Non-quality assured ACT Dosage not listed	A (MA062, 2009-Dec-15) WHO prequalification
Lufanter^®^, Bliss Gvs Pharma Ltd, India	Artemether-lumefantrine 20/120 mg and 80/480 mg	Non-quality assured ACT	Approval at national level
Lumart^®^, Cipla Ltd, India	Artemether-lumefantrine tablet 20/120 mg Artemether-lumefantrine tablet 40/240 mg	QAACT (Quality-assured ACT) Dosage not listed	A (MA064, 2009-May-22) WHO prequalification
Malacur^®^, Elder pharmaceuticals LTD, India	Dihydroartemisinin-piperaquine tablet 40/320 mg	Non-quality assured ACT Brand known, manufacturer not listed	Approval at national level
Malanil^®^, Glaxosmithkline Group Of Companies, Canada	Atovaquone-proguanil tablet 250/100 mg	Non-artemisinin therapy	Unknown
Maloxine^®^, Gracure Pharmaceuticals Ltd, India	SP tablet 500/25 mg	Non-artemisinin therapy Brand known, manufacturer not listed	Unknown
Mephaquin^®^, Mepha Ltd, Switzerland	Mefloquine tablet 250 mg	Non-artemisinin therapy	Unknown
P-Alaxin^®^, Bliss Gvs Pharma Ltd, India	Dihydroartemisinin-piperaquine tablet 40/320 mg	Non-quality assured ACT	Approval at national level
Paludrine^®^, Astra Zeneca UK Limited, United Kingdom	Proguanil tablet 100 mg	Prophylaxis	B
Pharmasucam^®^, Madras Pharmaceuticals, India	Artesunate-amodiaquine 100/270 mg	Not listed, brand name and manufacturer unknown	Unknown
Quinimax^®^, Sanofi Aventis, Spain	Quinine 500 mg and 125 mg	Non-artemisinin therapy	Unknown
R-Lume^®^, Impact Healthcare Pvt Ltd, India	Artemether-lumefantrine tablet 80/480 mg	Not listed, brand name and manufacturer unknown	Unknown
Savarine^®^, AstraZeneca Ltd, France	Proguanil-chloroquine tablet 200/100 mg	Not listed	Unknown
Sharlum^®^, Sharon Big-medicine Ltd, India	Artemether-lumefantrine tablet 80/480 mg and 40/240 mg	Not listed, brand name and manufacturer unknown	Unknown
Surquina^®^, Laboratoire Innotech International, France	Quinine tablet 250 mg	Non-artemisinin therapy	Unknown

*A* classified product—listed on WHO prequalification list, *B* classified product—Stringent National Drug Regulatory Authority Registration letter/Marketing Authorization. ACT Watch Antimalarial Database: (http://www.actwatch.info/databases/antimalarial_survey_data/az) WHO prequalification. (http://apps.who.int/prequal/query/ProductRegistry.aspx).

**Figure 3:**
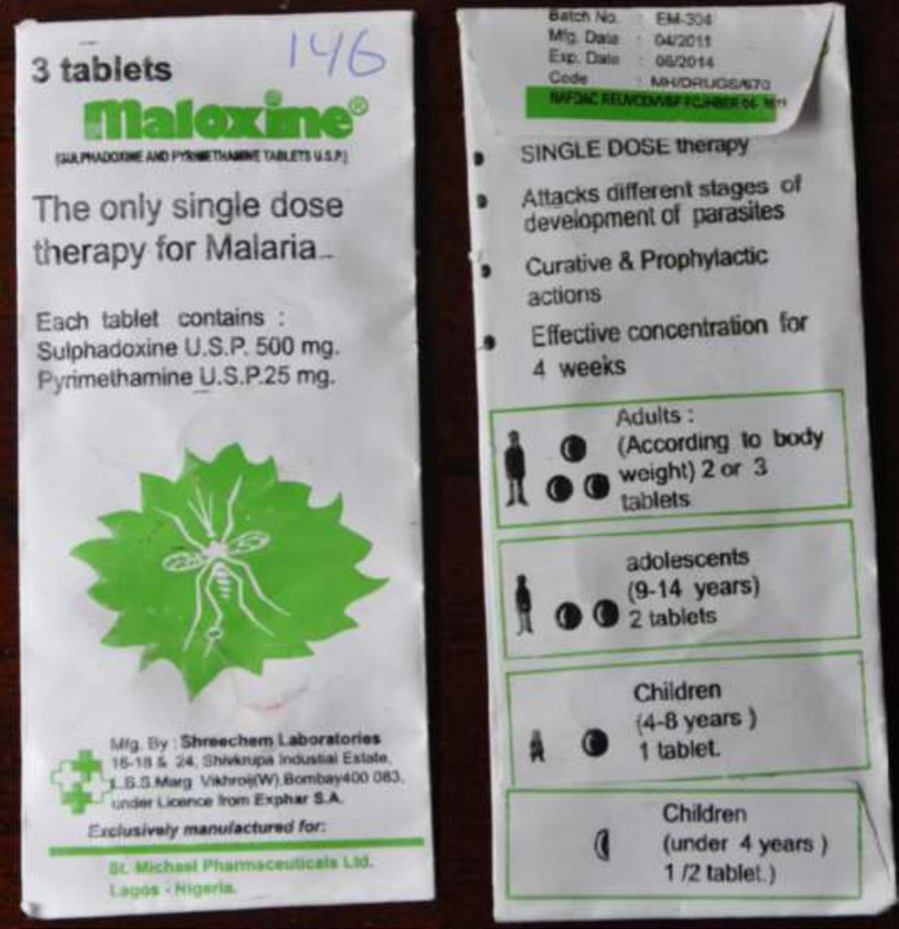
The suspect Maloxine^®^ sample, having failed semi-quantitative thin-layer chromatography. The manufacturer is wrong to allege that it is the “only single dose treatment of malaria”.

The other sample that failed TLC testing was SP 500/25 mg, Maloxine^®^ (Batch No. EM-304; Mfg. date: 04/2011; Exp. Date 08/2014; Code: MH/DRUGS/670 (Figure 3). No packaging analysis of the second failed sample was conducted, as results of previous attempts to collect genuine samples and batch information from the stated manufacturer for comparative assessment were unsuccessful.

There was no statistic significant difference (Fisher’s exact test, P = 0.311) in the number of poor-quality drugs collected by the Gabonese mystery shopper *versus* two European researchers (BJV and DK).

### High-performance liquid chromatography (HPLC) and artemisinin derivative screening test (ADST)

Both the ARST test (no colour produced) and HPLC (no peak on the spectra) analysis indicated the absence of API in the sample of Coartem^®^ batch no. F2261. In the suspect Maloxine^®^ sample batch no. EM-304 the stated APIs were detected, but the amount was approximately half the dose.

### Disintegration test

In total, 266 samples (62%) were tested for disintegration. Not all (n = 432) samples were tested because one disintegration test requires six tablets per test. Samples were tested per batch. One sample (0.4%) failed the disintegration test (Coartem^®^ sample that also failed the TLC and HPLC). The disintegration time of the Maloxine^®^ sample that failed the TLC test could not be tested, because the SP sample contains only three tablets in total. For detailed results of disintegration test per batch number see Additional file 5.

### Manufacturers and registration status

**Figure 4:**
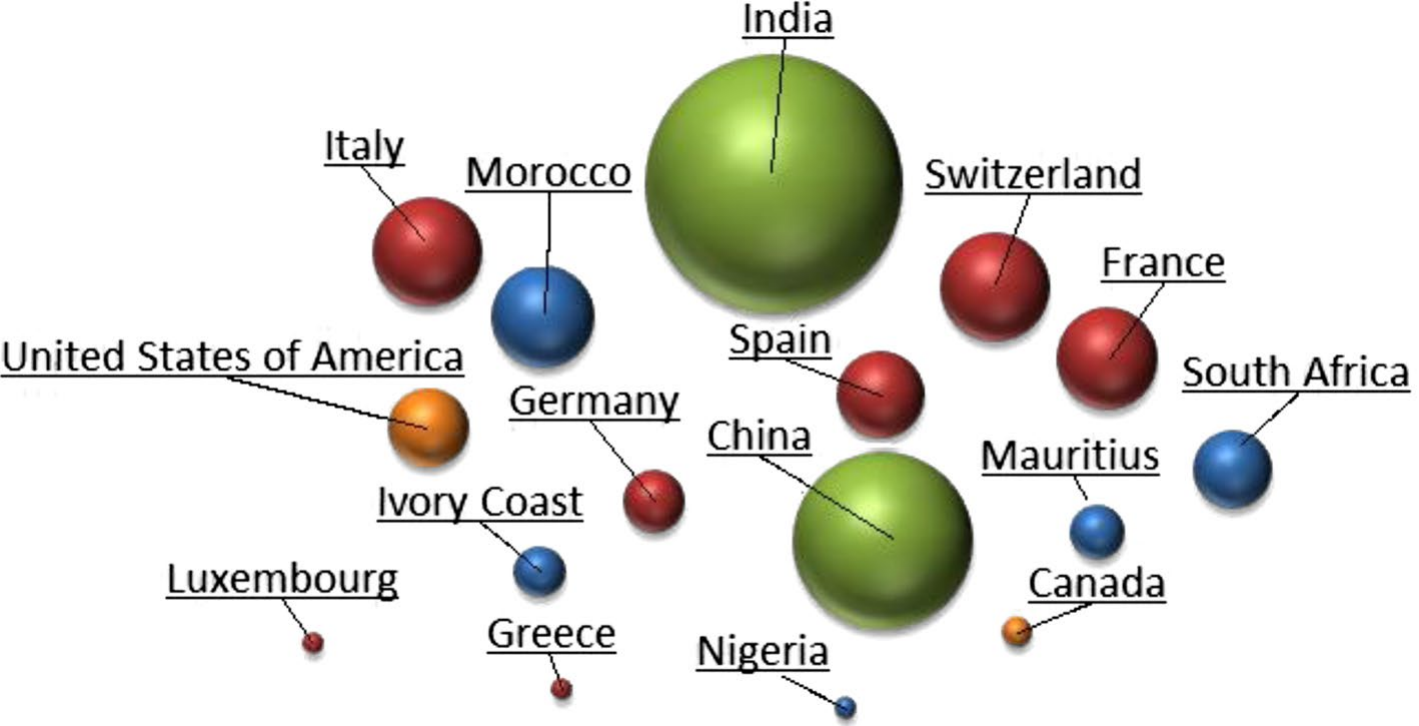
Stated origin of the collected samples. *Green* Asia, *Red* Europe, *Orange* North America.

Forty-three different brands were collected. Most packagings/accompanying leaflets of the drugs sampled stated the countries of manufacture to be India (n = 148, 34%) and China (n = 71, 16%) (Figure 4). Only five anti-malarial drug brands (87/432, 20% of samples) had obtained WHO prequalification: Coartem^®^, (n = 40, Novartis Pharma Ag); Lumart^®^, (n = 3, Cipla Ltd); Laritem^®^, (n = 3, IPCA Laboratories Ltd); Coarsucam^®^, (n = 22, Sanofi Aventis) and Artefan^®^, (n = 19, Ajanta Pharma Ltd). For many brands, the registration status in Gabon is unknown (Table 2).

### Questionnaire

In total, 209 adults randomly selected from street and market crowds participated in the survey [47]. As a rule of thumb, every fifth passer-by was approached to participate in the survey. Forty-one participants were interviewed at the market in Lambaréné, 98 at the market Mont Bouët in Libreville and 70 at PK8 (transport hub) in Libreville. For 203 people, data were complete; 92% (187/203) of the interviewed people purchased their anti-malarial drugs in a pharmacy, 3.4% in a hospital, 3.4% in a dispensary and 1% (2/203) on the market. Some 51% of the people reported to have a CNAMG (La Caisse Nationale d’Assurance Maladie et de Garantie Sociale du Gabon) health insurance, which means they receive anti-malarial drugs for free in CNAMG affiliated and licensed pharmacies. For detailed results of the questionnaire, see Additional file 6.

### Costs of this study

The total cost of this study [29] were: €7,578 including the purchase of samples, shipment of samples, transportation costs of the researchers and chemical analysis (GPHF Minilab™ = 4,491, €40, HPLC = in kind contribution), but excluding personnel costs and article-processing charges (Additional file 2).

## Discussion

This study represents the first systematic and nationwide field survey of anti-malarial drug quality in Gabon. The results are reassuring, as it has been demonstrated that poor-quality anti-malarial drugs were uncommon—only 0.5% of all samples failed the chemical analysis (TLC) and only one sample (AL, Coartem^®^) could be classified as falsified. The other sample (SP, Maloxine^®^) was considered as sub-standard as it contained half of the stated dose. The Coartem^®^ AL 20/120 mg sample that failed chemical testing was collected in a small pharmacy in Bitam, a town close to the border with Cameroon. This sample is a known fake [44]. In Cameroon, it was found that a large proportion of samples contained either no active ingredient, insufficiently active ingredient, the wrong ingredient, or unknown/unidentifiable ingredient(s) [5]. The authors do not know how this falsified sample ended up for sale in the pharmacy; probably this pharmacy purchased the drugs not via the regular route (the central pharmacy in Libreville), but via drug traders.

Reassuring was that no oral artesunate monotherapy was found. The results of this study are in strong contrast with findings from neighbouring countries [12], where high prevalence of poor-quality anti-malarial drugs have been reported. Using a randomized sampling collection method, fewer poor-quality anti-malarial drugs were identified, compared to previous reports using convenience-sampling methods. A systematic review in 2014 showed that only 5% (six) of 130 published reports included evidence for randomization of sample location selection [12].

The findings of the present study emphasize the need for randomized and reliable sampling methods in order to obtain reliable information on anti-malarial drug quality in a country. It also underpins the notion that there are important caveats to accurately estimate the prevalence and distribution of poor-quality anti-malarials, and that the problem may not be as universally massive as is suggested in the literature [12].

The low proportion of failures could also be explained by the fact that in Gabon, drugs importation and provision to pharmacies may be subject to better-enforced import regulation compared to other African countries. Theoretically (only, because income is unevenly distributed in Gabon), the average Gabonese resident may be more affluent (Gabon is an upper-middle income country), and thus have easier access and confidence to pharmacies for drug purchase.

A substantial amount of samples (brands) were not WHO-prequalified but registered at national level and refunded by the CNAMGS in Gabon [48, 49]. Registration of anti-malarials with no internationally regulatory clearance at national level may reflect a weakness in the regulatory system of Gabon.

### Strengths and limitations of study

The sample size in this study is appropriate to make generalizations about the quality of anti-malarial drugs in Gabon. Although the samples analysed in the current study were only collected from pharmacies (and not from markets, street vendors, etc.), the results arguably represent the situation in Gabon. In the experience of the authors and researchers at CERMEL, and as confirmed by the questionnaire results, it is known that most residents purchase their medicines in pharmacies or hospitals. Moreover, 92% of the interviewed people in this study reported to buy their drugs in a pharmacy, although having given ‘socially desirable answers’ cannot be excluded from this questionnaire. Another limitation of the street questionnaire is that it was conducted in only two cities: Lambaréné and the capital, Libreville. Therefore, these results may differ for other areas of Gabon.

Other strengths of this study are the randomized design and the use of a mystery shopper for the collection of samples [50]. The 11 surveyed cities/towns cover the majority of the relatively small population of Gabon. As can be seen on the map, towns are not evenly distributed across the country; most people live in the west, as the east of Gabon comprises large inaccessible tropical forest areas. All border areas were also covered in this survey: Equatorial Guinea (Libreville and Oyem), Cameroon (Bitam and Oyem), Congo (Franceville, Mouila).

The GPHF Minilab^®^ (semi-quantitative layer chromatography) was used to evaluate the quality of anti-malarial drugs. This method does not require specialist training, is simple to use, rapid, robust, reproducible, relatively inexpensive (HPLC of all the samples costs approximately €14,000), and has successfully detected poor-quality drugs before [37, 38, 51–55]. However, it is a screening tool able to give qualitative results as the sensitivity of the method is limited [56]. This means that false negatives might be present. A study conducted by the WHO in six African countries [22] compared the outcomes of quality control laboratory testing and the GPHF-Minilab^®^ screening. It was shown that Minilab screening detected only approximately one in three non-compliant samples. Furthermore, Minilab screening gave some false positive results: 6 of 99 ACT samples (6%) and 1 of 92 SP samples (1%) failed in GPHF-Minilab^®^ screening, but complied with all specifications in quality control laboratory testing. Thus, it can only reliably detect grossly sub-standard samples and therefore should not be used as an independent testing resource or provide quantitative data except in conjunction with a laboratory capable of more sensitive techniques, e.g., HPLC [52].

In this study, the two failed samples were analysed at the reference laboratory based at the London School of Hygiene and Tropical Medicine (LSHTM). Unfortunately, funds were insufficient to analyse all the ‘passed’ samples with HPLC to get a quantitative assessment of samples. However, even if the results underestimate the prevalence with a hypothetical factor 3, the prevalence of poor-quality drugs would be around 1.5%, which is still low (although significant in terms of morbidity and mortality). Thus, the GPHF Minilab^®^ can be useful as a screening tool of anti-malarial drugs through the use of semi-quantitative tests while results of a more thorough and confirmatory laboratory tests are awaited (e.g., mass spectrometry (FI-GRSA-MS) and HPLC) [57].

## Conclusion

Poor-quality anti-malarials are, according to described findings, uncommon in Gabon. Nevertheless, the Gabonese health authorities should lead an effort to improve regulatory requirements and consolidate regulatory functions, as recommend by WHO. It could also consider publishing and disseminating granted licences for drug manufacturers to improve the regulation of imported drugs.

## Additional files

Additional file 1: The medicine regulatory system in Gabon. This paragraph describes in brief the medicine regulatory system in Gabon. Additional file 2: Study costs. To increase transparency and reproducibility, the costs of this field survey are published. These include for example the laboratory costs, collection of the samples and travel expenses. Additional file 3: CERMEL Scientific Review Committee’s study protocol assessment. The assessment of the Scientific Review Committee of the study protocol. Additional file 4: Sample collection. The case record form or sample collection form depicts all the information collected during the sampling process. Additional file 5: Results of the disintegration test. This documents shows the detailed results of the disintegration tests. Additional file 6: AMQUAL questionnaire results. This documents summarizes the results of the street survey conducted.

## Abbreviations

ACT: artemisinin-based combination therapy
ADST: artemisinin derivative screening test
AL: artemether-lumefantrine
AMC: Academic Medical Center
API: active pharmaceutical ingredient
AS + AQ: artesunate + amodiaquine
CERMEL: Centre de Recherches Médicales de Lambaréné
CNAGMS: La Caisse Nationale d’Assurance Maladie et de Garantie Sociale du Gabon
ERP: expert review panel
FDA: US Food and Drug Administration
GMP: good manufacturing practices
GPHF: the global pharma health fund
HPLC: high-performance liquid chromatography
IMPACT: international medical products anti-counterfeiting taskforce
INN: international non-proprietary name
MEDQUARG: Medicine Quality Assessment Reporting Guidelines
MRA: Medicine Regulatory Agency
Ph. Eur.: European Pharmacopoeia
PQM: promoting the quality of medicines
QC: quality control
SOP: standard operating procedure
SP: sulfadoxine-pyrimethamine
TLC: semi-quantitative thin-layer chromatography
USP: US Pharmacopeia
UV: ultraviolet-visible
WHO: World Health Organization
WWARN: WorldWide Antimalarial Resistance Network
