# Supplementary material for: Assessing the quality of anti-malarial drugs from Gabonese pharmacies using the MiniLab®: a field study
Source: Malar J. 2015 Jul 15;14:273. doi: 10.1186/s12936-015-0795-z (PMC4501108; doi:10.1186/s12936-015-0795-z)
Supplement: Additional file 1: — The medicine regulatory system in Gabon. This paragraph describes in brief the medicine regulatory system in Gabon. [file 12936_2015_795_MOESM1_ESM.doc]

**Supplementary File 1.**

*The medicine regulatory system in Gabon*

In Gabon, a department within the Ministry of Health is responsible for the regulation of medicines. This national medicines regulatory authority (NMRA) is responsible for market authorization, licensing & import control, pharmocovigilance and control of promotion. For market authorization in Gabon, a brief description of required dossier components is available, but there is no guideline for applicants nor a SOP for assessment. There is an unofficial understaffed advisory committee and no external assessors. Market authorisation is mainly an administrative review and there are few requirements for generics. Important products need market authorisation, but there are some exceptions. The NMRA authorizes imports for specific needs without official delegation and without a defined procedure. Medicine donations and export are not regulated. The Division of Public Health Inspectorate is responsible for quality control, but due to lack of staff and resources this is not functioning; thus, external labs are used and plans to set up a Quality Control Laboratory in 2010 are not effectuated yet. There is no anti-counterfeiting programme or product quality monitoring and samples are not tested. A pharmacovigilance system does not exists; however, a draft reporting form exists.
