# Supplementary material for: Assessing the quality of anti-malarial drugs from Gabonese pharmacies using the MiniLab®: a field study
Source: Malar J. 2015 Jul 15;14:273. doi: 10.1186/s12936-015-0795-z (PMC4501108; doi:10.1186/s12936-015-0795-z)
Supplement: Additional file 2: — Study costs. To increase transparency and reproducibility, the costs of this field survey are published. These include for example the laboratory costs, collection of the samples and travel expenses. [file 12936_2015_795_MOESM2_ESM.doc]

**Supplementary File 2.**

*Study costs*

*Receipts are available for 99,7% of the total spending.*

*Total amount for which no (copy of) receipt is available: 10,21 €*

**Table. Summary of research costs of field survey in Gabon (including chemical analysis, excluding personnel and article-processing charge (±1720 euro))**

| **Specification** | **Amount in CFA-franc*** | **Amount in euro (€ )** | **Percentage of total costs** |
| --- | --- | --- | --- |
| Antimalarial drugs  *(432 samples)* | 1,619,572 CFA | 2468,86 € | 32.6% |
| GPHF Minilab™ | n/a | 4491,40 € | 59.3% |
| HPLC analysis (LSHTM) | - | In kind contribution | - |
| Transport costs** | 201,100 CFA | 306,55 € | 4.0% |
| Hotel costs *** | 122,300 CFA | 186,43 € | 2.5% |
| Other costs | 16,600 CFA | 25,30 € | 0.3% |
| Personnel costs | NS | NS | NS |
| ***Total costs*** |  | *7578,54 €* | *100%* |

* CFA= Communautés Financières d'Afrique (1 € = 656 CFA) ; NS=not specified

**Transport expenses for 3 fieldworkers and 2 researchers

***Hotel expenses for 2 researchers
