# Supplementary material for: Assessing the quality of anti-malarial drugs from Gabonese pharmacies using the MiniLab®: a field study
Source: Malar J. 2015 Jul 15;14:273. doi: 10.1186/s12936-015-0795-z (PMC4501108; doi:10.1186/s12936-015-0795-z)
Supplement: Additional file 3: — CERMEL Scientific Review Committee’s study protocol assessment. The assessment of the Scientific Review Committee of the study protocol. [file 12936_2015_795_MOESM3_ESM.pdf]

ASSESSMENT SHEET
SCIENTIFIC REVIEW COMMITTEE OF THE CERMEL, ALBERT SCHWEITZER HOSPITAL

**SRC-Number**	**2013.11**
**Principal Investigator**	**B.J. Visser**
**Corresponding Investigator**	**Martin Grobusch**
**Date of Submission**	**2013-10-03**
**Title**	**Antimalarial drug quality in Gabon**
**Acronym**	**AMQUAL**

**Overall Assessment**	**Score**<br>**Ok, n/a, clarification required**
Scientific quality	OK
Scientific interest	OK
Ethical considerations	OK
Feasibility of project	OK
Relationship to MRU research policy	OK
Relationship to national research policy	OK
Other issues	none
**Decision:**	**X Accepted without remarks**
	**□ Accepted with remarks**
	**□ Resubmit**
	**□ Rejected**

The investigators describe an important problem of international health sciences which has attracted considerable interest over the past decade. The SRC is grateful for the submission of the adapted documents and clarifications.

Documents required:

- □ **Final Protocol**
- □ **Informed Consent Form**
- □ **Ethics Approval**
- □ **Additional documents:**

Name and Signature: **Date:**

2013-10-18
