# Supplementary material for: Assessing the quality of anti-malarial drugs from Gabonese pharmacies using the MiniLab®: a field study
Source: Malar J. 2015 Jul 15;14:273. doi: 10.1186/s12936-015-0795-z (PMC4501108; doi:10.1186/s12936-015-0795-z)
Supplement: Additional file 4: — Sample collection. The case record form or sample collection form depicts all the information collected during the sampling process. [file 12936_2015_795_MOESM4_ESM.doc]

# Confidential

| **AMQUAL** |
| --- |

**Sample Collection Form**

| **Antimalarial drug quality in Gabon**  **Protocol number: Version 1.1**  **Study approval number: 2013.11** |
| --- |

**Date**  | |

*d d m m y y y y*

**Name collector:** 

**Phone number:** 

**Department:** 

**Centre:** 

*Principal Investigator: Dr. B.J. Visser*

*Albert Schweitzer Hôpital*

*Centre de Recherches Médicales de Lambaréné*

| **Site information** | | **Date:** | |  |
| --- | --- | --- | --- | --- |
| 1. | Country/Province | Gabonese Republic/……………………………. | | |
| 2. | Name of survey site  (e.g. village, city) |  | | |
| 3. | GPS location + no. |  | | |
| 4. | Address |  | | |
| 5. | Public vs. Private  Type of outlet: | □ Public □ Private  □ Hospital □ Clinic □ Pharmacy  □ Public dispensary □ NGO facility  □ Wholesaler □ Retail outlet  □ Informal market, please describe:  □ Other, please describe: | | Photo:  Yes   No  |
| **Drug sample** | |  | |  |
| 6. | Commercial name of the product |  | | |
| 7. | International Non-Proprietary Names of active ingredients |  | | |
| 8. | List of excipients |  | | |
| 9. | Dosage form (e.g. tablet, capsule) | □ Tablet □ Other, specify……… | | |
| 10. | Strength per unit dose (e.g. mg/tablet): | ………… / …………/ …………(unit) | | |
| 11. | Type and packaging material (e.g. strips/bottle) | □ Strips □ Bottle □ Other, specify……… | | |
| 12. | Quantity collected per sample, with specification of the package size | Taken in original package:   Taken from bulk container:  | | No: |
| 13. | Batch number or Lot Number |  | | |
| 14. | Manufacturing date & expiry date |  |  | |
| 15. | Country and postal address of manufacturer | ………………………………………………………………………………………………………………………………  .................................................................................................................................................................................. | | |
| 16. | Regulatory status of product in the country (i.e. registered, unregistered or other and, if registered, marketing authorization holder and number) | ........................................................................................................................................................................................................................................................................... | | |
| 17. | Any other comments | **Price:**………………………………………………………………………………………………………………………………………………………………………………………………………………………………………………………… | | |

| Signature |
| --- |
| Initials of collector |
| Date signed:     *d d m m y y y y*  Report reviewed by:    Name researcher:  Date data entry:     *d d m m y y y y* |
