# Supplementary material for: Assessing the quality of anti-malarial drugs from Gabonese pharmacies using the MiniLab®: a field study
Source: Malar J. 2015 Jul 15;14:273. doi: 10.1186/s12936-015-0795-z (PMC4501108; doi:10.1186/s12936-015-0795-z)
Supplement: Additional file 5: — Results of the disintegration test. This documents shows the detailed results of the disintegration tests. [file 12936_2015_795_MOESM5_ESM.doc]

**Supplementary File 5.**

*Results of the disintegration test*

| **Batch** | **Sample no’s** | **Type of tablet** | **Active Pharmaceutical Ingredient(s)** | **Pass-fail** |
| --- | --- | --- | --- | --- |
| **162** | 036, 005, 273 | Uncoated | Artemether-lumefantrine | Pass |
| **CF-41** | 016, 256, 227, 191, 406 | Film coated | Artemether-lumefantrine | Pass |
| **LT-80** | 019, 350, 370 | Film coated | Artemether-lumefantrine | Pass |
| **X1638** | 094, 426, 204, 300, 179 | Uncoated | Artemether-lumefantrine | Pass |
| **LXA3001A** | 203, 076, 045 | Uncoated | Artemether-lumefantrine | Pass |
| **X1631** | 420, 131 | Uncoated | Artemether-lumefantrine | Pass |
| **X1639** | 230, 312, 161 | Uncoated | Artemether-lumefantrine | Pass |
| **X1673** | 341, 381, 288 | Uncoated | Artemether-lumefantrine | Pass |
| **X1640** | 269, 359, 411 | Uncoated | Artemether-lumefantrine | Pass |
| **X1636** | 248, 279 | Uncoated | Artemether-lumefantrine | Pass |
| **X1637** | 400, 365 | Uncoated | Artemether-lumefantrine | Pass |
| **F1078** | 160,403, 306, 226 | Uncoated | Artemether-lumefantrine | Pass |
| **F0840A** | 298, 284, 195 | Uncoated | Artemether-lumefantrine | Pass |
| **F0840** | 244, 339 | Uncoated | Artemether-lumefantrine | Pass |
| **LT-79** | 038, 397 | Film coated | Artemether-lumefantrine | Pass |
| **LT-75** | 055, 111 | Film coated | Artemether-lumefantrine | Pass |
| **620522** | 222, 173, 188, 379 | Unknown | Artemether-lumefantrine | Pass |
| **GE130612** | 241, 331 | Uncoated^#^ | Artemether-lumefantrine | Pass |
| **KW3057** | 235, 088 | Uncoated | Artemether-lumefantrine | Pass |
| **LT-76** | 364 | Film coated | Artemether-lumefantrine | Pass |
| **LT-86** | 184 | Film coated | Artemether-lumefantrine | Pass |
| **LT-81** | 211 | Film coated | Artemether-lumefantrine | Pass |
| **LT-68** | 099 | Film coated | Artemether-lumefantrine | Pass |
| **CWY023005** | 254, 245, 153, 021, 181, 081, 162 | Unknown | Artemether-lumefantrine | Pass |
| **CWY023009** | 392, 265, 319, 218 | Unknown | Artemether-lumefantrine | Pass |
| **13052** | 014, 326 | Uncoated | Artemether-lumefantrine | Pass |
| **13435** | 220, 237, 168 | Uncoated | Artemether-lumefantrine | Pass |
| **GE130103** | 228 | Uncoated | Artemether-lumefantrine | Pass |
| **111189** | 089 | Uncoated | Artemether-lumefantrine | Pass |
| **GE2018** | 121 | Uncoated | Artemether-lumefantrine | Pass |
| **GE130309** | 278 | Uncoated | Artemether-lumefantrine | Pass |
| **LT-71** | 290 | Film coated | Artemether-lumefantrine | Pass |
| **LT-67** | 148 | Film coated | Artemether-lumefantrine | Pass |
| **AP-007** | 171 | Uncoated | Artemether-lumefantrine | Pass |
| **E-3501** | 001 | Uncoated | Artemether-lumefantrine | Pass |
| **F2261** | 383 | Uncoated | Artemether-lumefantrine | Fail |
| **C0121J** | 221 | Uncoated | Artemether-lumefantrine | Pass |
| **CF-39** | 070 | Film coated | Artemether-lumefantrine | Pass |
| **C0501I** | 002 | Uncoated | Artemether-lumefantrine | Pass |
| **X1672** | 067 | Uncoated | Artemether-lumefantrine | Pass |
| **E12556** | 086 | Uncoated | Artemether-lumefantrine | Pass |
| **CWY023006** | 106, 142, 026 | Uncoated | Artemether-lumefantrine | Pass |
| **X1611** | 384 | Uncoated | Artemether-lumefantrine | Pass |
| **X1665** | 044 | Uncoated | Artemether-lumefantrine | Pass |
| **X1651** | 015 | Uncoated | Artemether-lumefantrine | Pass |
| **X1629** | 140 | Uncoated | Artemether-lumefantrine | Pass |
| **X1630** | 033 | Uncoated | Artemether-lumefantrine | Pass |
| **X1604** | 107 | Uncoated | Artemether-lumefantrine | Pass |
| **CWY023008** | 063, 297 | Unknown | Artemether-lumefantrine | Pass |
| **A103021** | 177, 223 | Film coated | Artemether-lumefantrine | Pass |
| **159** | 051, 212 | Uncoated | Quinine | Pass |
| **160** | 202 | Unknown | Quinine | Pass |
| **10564** | 323 | Film coated | Quinine | Pass |
| **161** | 432 | Unknown | Quinine | Pass |
| **20849** | 037 | Film coated | Quinine | Pass |
| **No batch number** | 900 | Unknown | Quinine | Pass |
| **KD-234** | 386 | Unknown | Quinine sulphate | Pass |
| **10565** | 410,434 | Film coated | Quinine sulphate | Pass |
| **10261** | 087 | Coated | Quinine sulphate | Pass |
| **18924** | 003 | Film coated | Quinine hydrochloride | Pass |
| **15553** | 108 | Film coated | Quinine hydrochloride | Pass |
| **1601** | 193 | Film coated | Quinine hydrochloride | Pass |
| **12** | 018 | Film coated* | Quinine hydrochloride | Pass |
| **1602** | 209, 313, 152 | Film coated | Quinine hydrochloride | Pass |
| **1603** | 075, 261, 280 | Film coated | Quinine hydrochloride | Pass |
| **J3** | 409, 158, 396, 208, 264 | Film coated | Quinine hydrochloride | Pass |
| **22668** | 344, 054 | Film coated | Quinine hydrochloride | Pass |
| **J1** | 423, 321, 098, 342, 194 | Film coated | Quinine hydrochloride | Pass |
| **19784** | 027, 139, 074 | Film coated | Quinine hydrochloride | Pass |
| **23185** | 240, 093 | Film coated | Quinine hydrochloride | Pass |
| **121201** | 207, 418, 387, 310, 157, 175, 431, 236 | Coated | Dihydroartemisinin-piperaquine phosphate- trimethoprim | Pass |
| **120101** | 338, 041 | Coated | Dihydroartemisinin-piperaquine phosphate- trimethoprim | Pass |
| **130101** | 170, 373 | Uncoated | Dihydroartemisinin-piperaquine phosphate- trimethoprim | Pass |
| **SM0063025E** | 275, 219, 189, 317 | Film coated | Dihydroartemisinin-piperaquine phosphate | Pass |
| **120834** | 311, 124, 398, 149, 408, 092, 078 | Uncoated | Dihydroartemisinin-piperaquine phosphate | Pass |
| **SM0063011E** | 069, 205, 378 | Film coated | Dihydroartemisinin-piperaquine phosphate | Pass |
| **SM0063013E** | 293, 031, 105, 004, 147 | Film coated | Dihydroartemisinin-piperaquine phosphate | Pass |
| **SM0062021E** | 178 | Coated | Dihydroartemisinin-piperaquine phosphate | Pass |
| **121119** | 010 | Unknown | Dihydroartemisinin-piperaquine phosphate | Pass |
| **120835** | 112 | Unknown | Dihydroartemisinin-piperaquine phosphate | Pass |
| **0738** | 091 | Film coated | Dihydroartemisinin-piperaquine phosphate | Pass |
| **SM0063019E** | 345, 390 | Film coated | Dihydroartemisinin-piperaquine phosphate | Pass |
| **SM0063012E** | 415, 295 | Film coated | Dihydroartemisinin-piperaquine phosphate | Pass |
| **121023** | 025, 302, 336 | Film coated | Dihydroartemisinin-piperaquine phosphate | Pass |
| **120833** | 057, 274 | Film coated | Dihydroartemisinin-piperaquine phosphate | Pass |
| **SM0063016E** | 101 | Film coated | Dihydroartemisinin-piperaquine phosphate | Pass |
| **110** | 243, 337, 289, 165 | Unknown | Artesunate-SP | Pass |
| **2563** | 428, 388, 185 | Uncoated^#^ | Artesunate-SP | Pass |
| **138** | 416, 301, 013, 368, 084, 394 | Unknown | Artesunate-SP | Pass |
| **151** | 144, 372, 047, 425, 354, 119, 183 | Unknown | Artesunate-SP | Pass |
| **143** | 285, 073, 353 | Unknown | Artesunate-SP | Pass |
| **123** | 154, 363, 028 | Unknown | Artesunate-SP | Pass |
| **M121069-2** | 266, 324, 187 | Film coated | Artesunate-amodiaquine | Pass |
| **050311** | 155, 250, 012 | Unknown | Artesunate-amodiaquine | Pass |
| **041211** | 412, 065, 433, 210, 244 | Unknown | Artesunate-amodiaquine | Pass |
| **5442** | 052, 180, 252, 113, 347, 022 | Unknown | artesunate-amodiaquine | Pass |
| **5349** | 129 | Unknown | Artesunate-amodiaquine | Pass |
| **2344** | 399,083,213,122 | Unknown | Artesunate-amodiaquine | pass |
| **135014** | 263, 104, 292, 167, 414 | Unknown | Artesunate-mefloquine | Pass |
| **20192** | 327, 247, 090, 272, 393, 422, 199, 040 | Unknown | Sulphadoxine-Pyrimethamine | Pass |
| **TE-4255** | 271, 159, 085 | Unknown | Sulphadoxine-Pyrimethamine | Pass |
| **TE-4223** | 246, 343, 216, 287 | Unknown | Sulphadoxine-Pyrimethamine | Pass |
| **TE-4314** | 072, 356, 135, 046, 117, 192 | Unknown | Sulphadoxine-Pyrimethamine | Pass |
| **Z9971** | 030, 136, 024, 430, 077 | Unknown | Sulphadoxine-Pyrimethamine | Pass |
| **B12011307** | 315, 360 | Unknown | Mefloquine | Pass |

***Samples disintegrated in time, but coating remained almost intact; #: Samples disintegrated in time, but appeared not dissolve: thick layer of sediment on the bottom, disintegration fluid stays clear; ˚: delivered in a plastic bag, no users information; ˠ: delivered as single tablet strip. No users information;**

Notes to the table: As can be seen from the data in table 3, several samples did disintegrate in time, but left the coating in the basket. All these samples were tested for a second time, with 0.1 M hydrochloride instead of water R as disintegration fluid. This time the coating disappeared during disintegration and passed the disintegration test.
