# Supplementary material for: Assessing the quality of anti-malarial drugs from Gabonese pharmacies using the MiniLab®: a field study
Source: Malar J. 2015 Jul 15;14:273. doi: 10.1186/s12936-015-0795-z (PMC4501108; doi:10.1186/s12936-015-0795-z)
Supplement: Additional file 6: — AMQUAL questionnaire results. This documents summarizes the results of the street survey conducted. [file 12936_2015_795_MOESM6_ESM.doc]

**Supplementary File 6.**

AMQUAL Questionnaire results

|  | **Male**  *(n= 115)* | **Female**  *(n=94)* |
| --- | --- | --- |
| **Age** (mean; min-max) | 29 (17-58) | 32 (16-63) |
| **Highest education** (highest)  *None*  *Primary school*  *Secondary school*  *Higher education (BAC/CAP)*  *University (BUT/licence)* | (n=115)  11 (9.5%)  22 (19.2%)  46 (40%)  23 (20%)  13 (11.3%) | (n=94)  9 (9.6%)  17 (18.1%)  23 (24.5%)  9 (9.5%)  6 (6.4%) |
| **Possessions** (multiple answers)  *Mobile phone*  *Freezer*  *Fridge*  *Television*  *House*  *Car* | (n=113)  113 (100%)  10 (9%)  19 (17%)  20 (18%)  12 (11%)  6 (5.3%) | (n=94)  92 (98%)  11 (12%)  23 (24%)  25 (27%)  6 (6.3%)  0 (0%) |
| **Smoking**  *Yes (average sig. per day)*  *None* | (n=100)  16 (16%) (mean: 5.4 sig.)  84 (84%) | (n=93)  1 (1%) (5-6 sig.)  92 (99%) |
| **Alcohol use**  *Yes*  *None* | (n=109)  27 (25%)  82 (75%) | (n=90)  14 (16%)  76% (84%) |
| **Marital status**  *Single*  *Married*  *Separated/Divorced*  *Widow* | (n=115)  59 (51%)  54 (47%)  2 (2%)  0 (0%) | (n=94)  42(45%)  49 (52%)  1 (1%)  2 (2%) |
| **Housing situation**  *Living alone*  *Living together*  *Number of children (median)*  *Number of roommates (mean)* | (n=114)  12 (11%)  102 (89%)  2 [0-29]  3 | (n=89)  2 (2%)  87 (98%)  2 [0-6]  3 |
| **Health insurance**  *Yes (CNAMGS)*  *None* | (n= 113)  60 (53%)  53 (47%) | (n=93)  45 (48%)  48 (52%) |
| **Number of malaria episodes**  1-2  3-4  >5 | (n=115)  14 (12.2%)  60 (52.2%)  41 (35.7%) | (n=93)  8 (8.6%)  49 (52.6%)  36 (38.7%) |
| **Last malaria episode**  *Last month*  *Last year*  *More than 1 year ago* | (n=113)  17 (15%)  29 (26%)  69 (59%) | (n=93)  17 (18%)  22 (24%)  54 (59%) |
| **Diagnosis of malaria**  *Self-diagnosis*  *Doctor*  *Traditional healer*  *Pharmacist*  *Pastor/Preacher* | (n=108)  35 (32.4%)  64 (59.2%)  4 (3.7%)  3 (2.8%)  2 (1.8%) | (n=94)  36 (38.2%)  48 (51%)  5 (5.3%)  4 (4.2%)  1 (1.1%) |
| **Use of prescribed drugs**  *Yes*  *No* | (n=100)  92 (92%)  8 (8%) | (n=78)  72 (92%)  6 (8%) |
| **Type of drugs**  *Antimalarial drugs*  *Traditional drugs (e.g. eboga)*  *Prayer* | (n=110)  105 (95%)  13 (12%)  32 (29%) | (n=90)  88 (98%)  15 (17%)  36 (40%) |
| **Buyer of drugs**  *Yourself*  *Family*  *Friends*  *Other* | (n=114)  80 (70%)  14 (12%)  19 (17%)  1 (1%) | (n=85)  72 (85%)  11 (13%)  2 (2%)  0 (0%) |
| **Place of purchase**  *Pharmacy*  *Market*  *Dispensary*  *Hospital*  *Traditional healer*  *Grocer*  *Family or friends*  *Other (e.g. street-vendor)* | (n= 111)  104 (93.7%)  1 (0.9%)  2 (1,8%)  4 (3.6%)  0 (0%)  0 (0%)  0 (0%)  0 (0%) | (n=92)  83 (90.2%)  1 (1.08%)  5 (5.4%)  3 (3.3%)  0 (0%)  0 (0%)  0 (0%)  0 (0%) |
